# Handheld portable device for delivering capped silver nanoparticles for antimicrobial applications

**DOI:** 10.1017/qrd.2024.9

**Published:** 2024-12-03

**Authors:** Kumar Naveen, Sandeep Bose, Chanbasha Basheer, Richard N. Zare, Elumalai Gnanamani

**Affiliations:** 1 Department of Chemistry, Indian Institute of Technology Roorkee, Roorkee 247667, India; 2 Department of Chemistry, Stanford University, Stanford, CA 94305, USA; 3 Chemistry Department, King Fahd University of Petroleum and Minerals, Dhahran 31261, Saudi Arabia

**Keywords:** Silver nanoparticles, water microdroplets, wound healing, handheld, portable

## Abstract

We describe a simple, cost-effective, green method for producing capped silver nanoparticles (Ag NPs) using a handheld portable mesh nebulizer. The precursor solution containing a 1:1 mixture of silver nitrate (AgNO_3_) and ligand (glycerol or sodium alginate) was sprayed using the nebulizer. The Ag NPs were generated in the water microdroplets within a few milliseconds under ambient conditions without any external reducing agent or action of a radiation source. The synthesized nanoparticles were characterized by using high-resolution transmission electron microscopy (HR-TEM), X-ray photoelectron spectroscopy (XPS), and X-ray diffraction analysis (XRD), which validated the formation of Ag NPs. The synthesized glycerate-capped silver nanoparticles (Ag-gly NPs) were used as a catalyst to show the oxidative coupling of aniline to form azobenzene products with a yield of up to 61%. Experiments conducted using Ag NPs produced in the droplets demonstrated more than 99% antibacterial activity when contacting *Escherichia Coli.* Our in-situ synthesis-cum-fabrication technique using a portable sprayer represents a viable alternative to the existing fiber or hydrogel-based antimicrobial wound healing.

## Introduction

Conventional strategies for the treatment of microbial wound infections require the use of disinfectants and antibiotics (Daeschlein, 2013). Regrettably, excessive amounts of antibiotics have led to the development of bacterial resistance (Ventola, 2015). This has become a threatening situation for humanity and a great challenge for the scientific world. Silver is well-established for its antimicrobial and anti-inflammatory properties (White, 2001). However, silver ions exhibited side effects like accumulation in normal tissues and organs by diffusing back and forth at the cellular level (Arvizo et al., 2012). Nanotechnology could potentially overcome these challenges by properly using silver metal as an antimicrobial agent, although care must be taken to avoid inhalation (Quadros and Marr, 2010).

Silver nanoparticles (Ag NPs) are usually prepared from Ag(I) by chemical routes and are considered less toxic than Ag(I) (Dong et al., 2021). In general, the preparative method takes a long time (Raveendran et al., 2003; Raveendran et al., 2006), demonstrates aggregation (Van Hyning and Zukoski, 1998), and involves toxic chemical reagents (Wang et al., 2023; Liu et al., 2020; Neumaier et al., 2021; Stewart et al., 2012), which could potentially damage tissues and organs. In addition, the clinical application requires biocompatibility and a ready-to-use methodology that will not only help the clinicians to administer the nanoparticles (NPs) on demand but also work as a fabrication tool to actively treat the wounds in a short time. Therefore, a simple, handheld, portable device that can work as an Ag NPs synthesis-cum-fabrication technique would be a welcome new tool.

Traditionally, silver nanoparticles were synthesized using reducing agents such as sodium borohydride (NaBH_4_) to reduce silver ions into silver nanoparticles (Van Hyning and Zukoski, 1998). Bare Ag NPs quickly undergo self-aggregation. To overcome self-aggregation, organic molecules have been utilized as capping agents, such as the introduction of long-chain carboxylates (Wang et al., 2023), long-chain thiols (Neumaier et al., 2021), cetyltrimethyl-ammonium bromide (Sakai et al., 2006), polymers like poly(N-vinyl-2-pyrrolidone) (Jharimune et al., 2021), and copolymers (Bockstaller et al., 2003) to obtain the NPs. To minimize the use of hazardous organic reagents, several other methods are available for the preparation of silver nanoparticles, for example, the biosynthesis of nanoparticles microorganisms such as using bacteria (Hebbalalu et al., 2013), yeasts (Mukherjee et al., 2001), fungi (Nasrollahzadeh et al., 2019), and *in vitro* biosynthesis using silver binding peptides (Naik et al., 2002). An eco-friendlier approach is to synthesize silver nanoparticles by simultaneously using water as a solvent, an environmentally benign reducing agent, and a non-toxic capping reagent (Yao et al., 2022). Although several methods are available for the synthesis of Ag NPs, most require reducing agents, higher temperatures, or photochemical procedures and long reaction times (Sun and Xia, 2002; Liu et al., 2020; Maretti et al., 2009; Wang et al., 2023; Pankongadisak et al., 2014; Simo et al., 2012).

Keeping in mind the clinical requirements and difficulties of Ag NPs synthesis, we have demonstrated a portable ultrasonic spray device that uses water microdroplets for an in-situ synthesis-cum-fabrication of medicinally active alginate and glycerol-capped Ag NPs within a few milliseconds under ambient conditions. These will not only fulfil the on-demand nanoparticle fabrication on the wounds as required by clinicians but also take care of the synthesis-related disadvantages. We were encouraged to try this water microdroplets-based Ag NPs preparation owing to the exceptional behavior of the water microdroplets at the interface. Water microdroplets were previously shown to accelerate the reaction rate and lead to the formation of unexpected products (Song et al., 2022; Jin et al., 2023; Vogel et al., 2020; Meng et al., 2023; Lee et al., 2018; Banerjee et al., 2017; Yan et al., 2016; Nandy et al., 2023; Bose et al., 2021; Satyabola et al., 2021; Ray Chowdhuri et al., 2021; Zhu et al., 2023), such as the production of gold nanostructures through the reduction of chloroauric acid (Lee et al., 2018). We believe our rapid NPs formation is due to such a redox reaction in the microdroplets, which proceeds in a very short time. Herein, we also demonstrated the antibacterial properties of our synthesized Ag NPs and showed their catalytic activities for oxidative coupling reactions of industrial importance.

## Results and discussion

### (a) Synthesis and characterization

**Figure 1.**
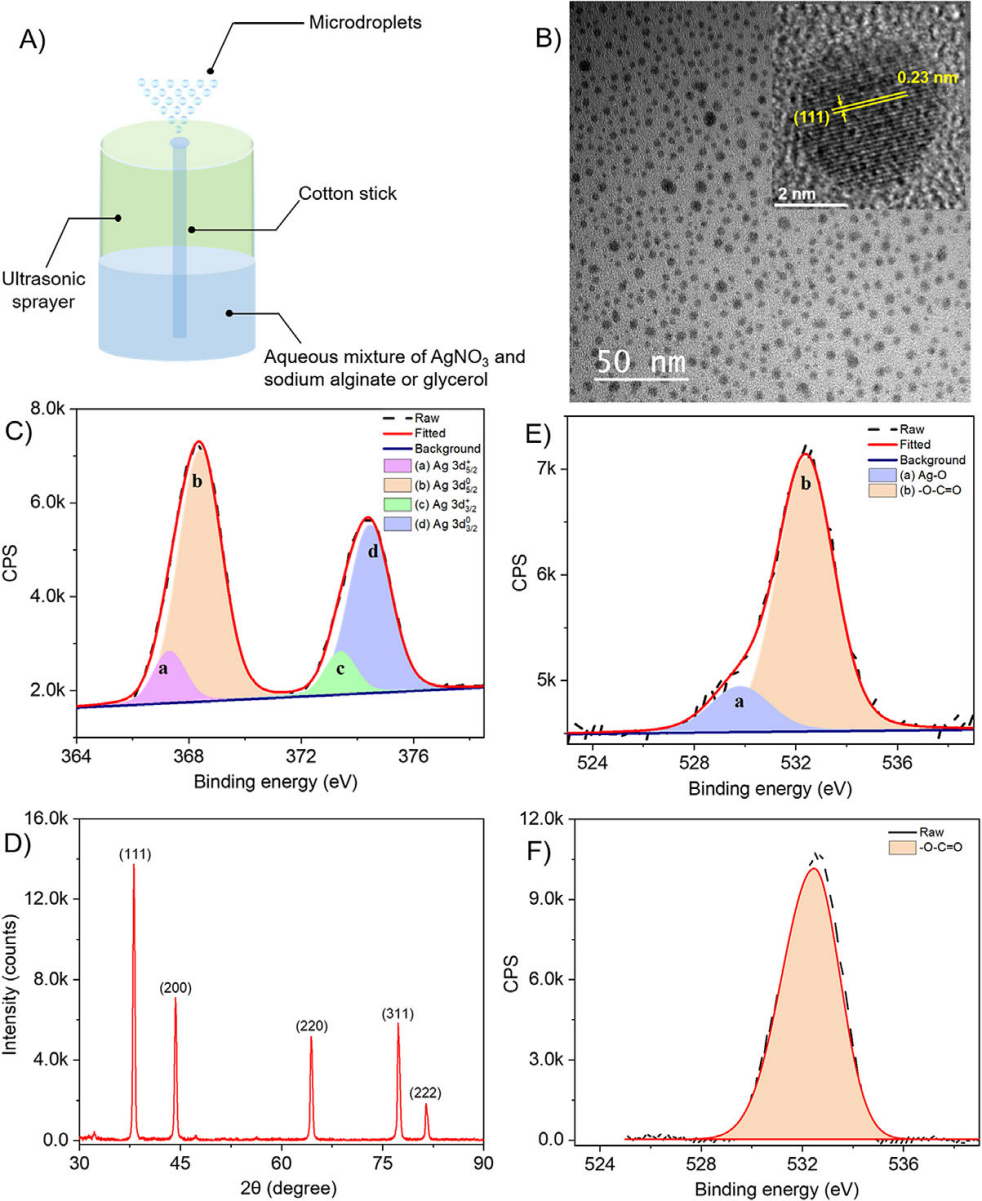
(A) Schematic of the ultrasonic device used for the preparation of Ag NPs. (B) Larger area TEM image of the Ag-gly NPs with the inset showing a crystalline lattice with a spacing of 0.23 nm due to (111) lattice plane of silver. (C) Deconvoluted XPS spectrum of Ag-gly NPs showing 3d features of Ag. (D) XRD features of Ag-gly NPs. (E) Deconvoluted O 1s spectrum of Ag-gly NPs. (F) O 1s spectrum of glycerol.

Figure 1A depicts the schematic of the experimental setup. AgNO_3_ (25 μmol) was dissolved in 1:1 (v/v) glycerol/water (3 mL) and the resulting mixture was sprayed using the mesh nebulizer spray device. The photographic image of the device-producing microdroplets is shown in Figure S1. The reaction product was collected in a vial and stored for further characterization. The mixture solution after being sprayed shows a brown color which was not noticed before spray, indicating the possible formation of Ag NPs (Figure S2). To confirm the formation of Ag NPs, the AgNO_3_ in glycerol-water solution was directly sprayed onto a TEM grid for 30 s. Figure 1B shows a large area TEM image of the Ag NPs. The picture depicts a high population of NPs formed during the process, which are mostly spherical. The inset in Figure 1B shows a lattice spacing of 0.23 nm originating from the (111) plane of silver (Ag) (Liu et al., 2023). Energy dispersive X-ray spectroscopy (EDS) measurement shows the presence of Ag (Figure S3). Previous reports show that glycerol and sodium alginate were used as reducing agents in synthesizing Ag NPs (Liu et al., 2020; Jahangir et al., 2023; Munir and Yesiloz, 2023). Consequently, we have performed the experiment even in the absence of glycerol and sodium alginate (Figure S4). We observed the formation of nanoparticles even in the absence of glycerol and sodium alginate suggesting the use of glycerol as a capping agent only. The silver nanoparticles are reduced by the electrons present in the microdroplets (Chen et al., 2024; Yuan et al., 2023; Chen et al., 2023; Zhao et al., 2022; Gong et al., 2022). The powder XRD pattern (Figure 1C) of the synthesized NPs demonstrates their crystalline structure. A characteristic splitting of peaks occurred at 2θ (i.e., 38.1°,44.3°, 64.4°, 77.3°, and 81.5°) as 111, 200, 220, 311, and 222 planes, respectively. The diffraction peak corresponding to the face-centered cubic (fcc) crystalline silver phase (JCPDS card number: 87–0717) is also clearly visible in the obtained pattern. Furthermore, the data agreed closely with previously reported literature values (Munir and Yesiloz, 2023).

We investigated X-ray photoelectron spectroscopy (XPS) to show the oxidation states of silver. Figure 1D depicts the XPS spectrum of silver upon NPs formation. A doublet feature at 368.2 eV and 374.3 eV corresponds to 3d_5/2_ and 3d_3/2_ peaks of metallic Ag (Sharma et al., 2018). The deconvolution of 3d_5/2_ and 3d_3/2_ peaks shows an additional doublet feature of low intensity at 367.5 eV and 373.5 eV due to Ag^+^. The emergence of Ag^+^ is possibly caused by the surface functionalization of the NPs with the glycerolate ion, which causes the surface oxidation of Ag by the formation of the Ag-O bond. To confirm the Ag-O functionalization at the surface, we analyze the O 1s peak of the glycerol before and after NPs formation. Before NPs formation, O 1s shows a feature at 532. 2 eV due to –O- C=O (Figure 1E) which is shifted to 532.4 eV upon the formation of NPs (Figure 1F). The O 1s peak is slightly shifted to higher binding energy after NPs formation. This shift could be from the enhanced electropositive character of carboxylate oxygen which arises as a result of sharing electron density with the attached Ag^+^ ion. An additional feature at binding energy 530.4 eV was observed. Such features were obtained when a metal oxide bond exists in the system. Hence, the peak at 530.4 eV could be attributed to the O-Ag bond in the system (Rodrigues et al., 2019; Bose et al., 2024). The C 1s feature of glycerol shows C-O and C=O peaks at 286.3 eV and 288.2 eV, which after NPs formation change to 286.5 eV and 288.4 eV, respectively (Figure S5). A similar shift of C 1s features toward higher binding energy upon NPs formation further supports our observation of surface functionalization.

To prove that our method of generating NPs is universal and can be employed for producing other materials, we synthesized alginate-capped Ag NPs as well as nickel nanoparticles. The characterization shown in Figure S6 confirms the formation of Ag NPs. The TEM image of the nickel nanoparticles is shown in Figure S7. These observations prove our device is not selective to metal or ligand and may be useful for producing other metallic NPs.

### (b) Enhancement of the rate of the reaction in microdroplets

**Figure 2.**
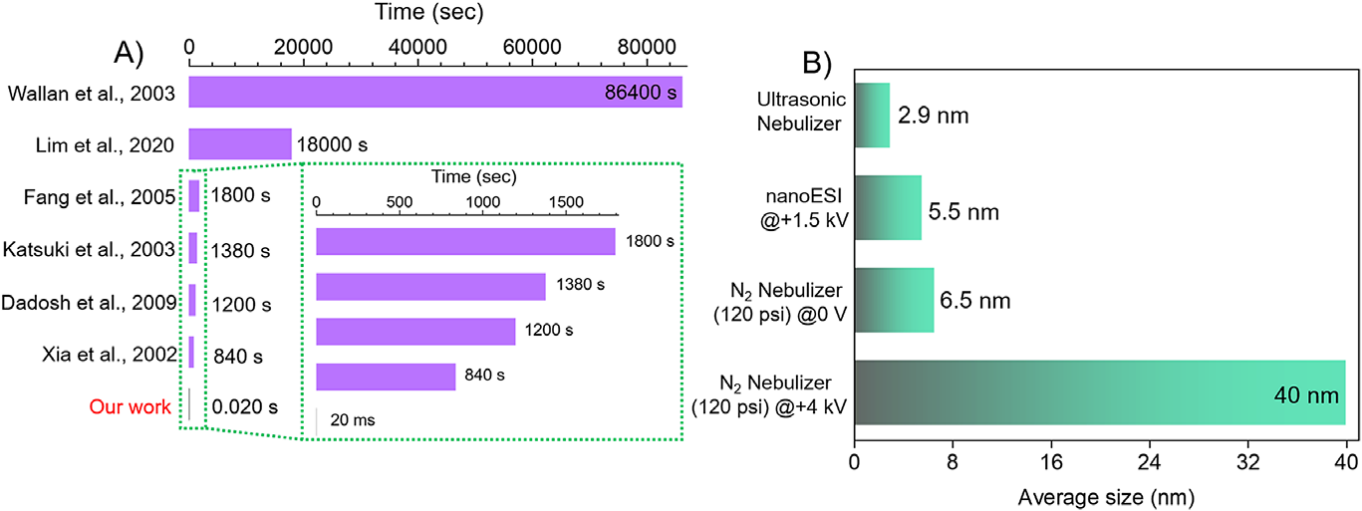
(A) Comparison of the time required for Ag NPs synthesis by our method with the others reported in the literature (Raveendran et al., 2003; Sun and Xia, 2002; Liu et al., 2020; Chen et al., 2023; Zhao et al., 2022; Gong et al., 2022). (B) The average size of the Ag NPs obtained by various microdroplet syntheses.

One of the advantages of droplet-based synthesis is that it requires an extremely small time scale up to the order of milliseconds to form nanoparticles. Numerous reports exist in the literature on the synthesis of Ag NPs but most of them require a minimum of 10 min or more (bottom-up approach) for the completion of reaction. We compare some of the literature-reported Ag NPs synthesis times with our method (Figure 2A). The time required for solution process syntheses varies from 14 min to 24 h whereas we require only milliseconds to form Ag NPs. Ag NPs synthesized by our method are 42,000, 60,000, 69,000, 90,000, 900,000, and 4,320,000 times faster than that reported by Sun and Xia (2002)), Dadosh (2009)), Katsuki and Komarneni (2003)), Fang et al. (Fang et al., 2005), Liu et al. (Liu et al., 2020), and Raveendran et al. (Raveendran et al., 2003), respectively (Figure 2A). Thus, it appears that the method we are describing is faster than any previous way of making silver nanoparticles.

### (c) Comparison with other microdroplet synthesis

There are various ways in which microdroplets can be generated and Ag NPs synthesis can be done other than our reported method. We have compared Ag NPs produced by our method with other microdroplet syntheses.

From Figure 2B, it appears that all the droplet synthesis techniques produce Ag NPs but only the mesh nebulizer produces smaller-sized Ag NPs. The average size of Ag NPs produced by our nebulizer is ~2.9 nm whereas nanoESI produces Ag NPs ~ 5.5 nm. Nitrogen as a nebulizing gas (120 psi) was also tested in the absence (0 V) and the presence of an applied voltage (+4 kV). In the absence, we observed an average size of ~6.5 nm whereas in the presence the Ag NPs size increases surprisingly to ~40 nm. Applying an external voltage seems to aggregate the Ag NPs resulting in larger sizes. The TEM image of the NPs produced by a different method and their respective size distribution is shown in Figure S8. We measured the median droplet size (d_50_) produced by each method and found our mesh nebulizer produces smaller size droplets than others (Figure S9). For mesh nebulizers, the droplets produced are fine and uniform as compared to N_2_-based nebulizers where the droplet generation is due to the high pressure exerted by the N_2_ gas. The pressure exerted on the droplet is non-uniform leading to less uniform and slightly bigger size microdroplets.

Small-sized Ag NPs are more effective in antibacterial properties owing to their greater surface area and better penetration into the bacterial cell membrane. The smaller the size of the Ag NPs, the smaller the value of minimum inhibitory concentration (MIC) and minimum bactericidal concentration (MBC) and therefore greater the antibacterial activity (Dong et al., 2019). Additional advantages include no use of any high electric field or N_2_ nebulizing gas for NPs synthesis which adds simplicity, safety, and affordability to our method.

### (d) Antibacterial and catalytic applications

**Figure 3.**
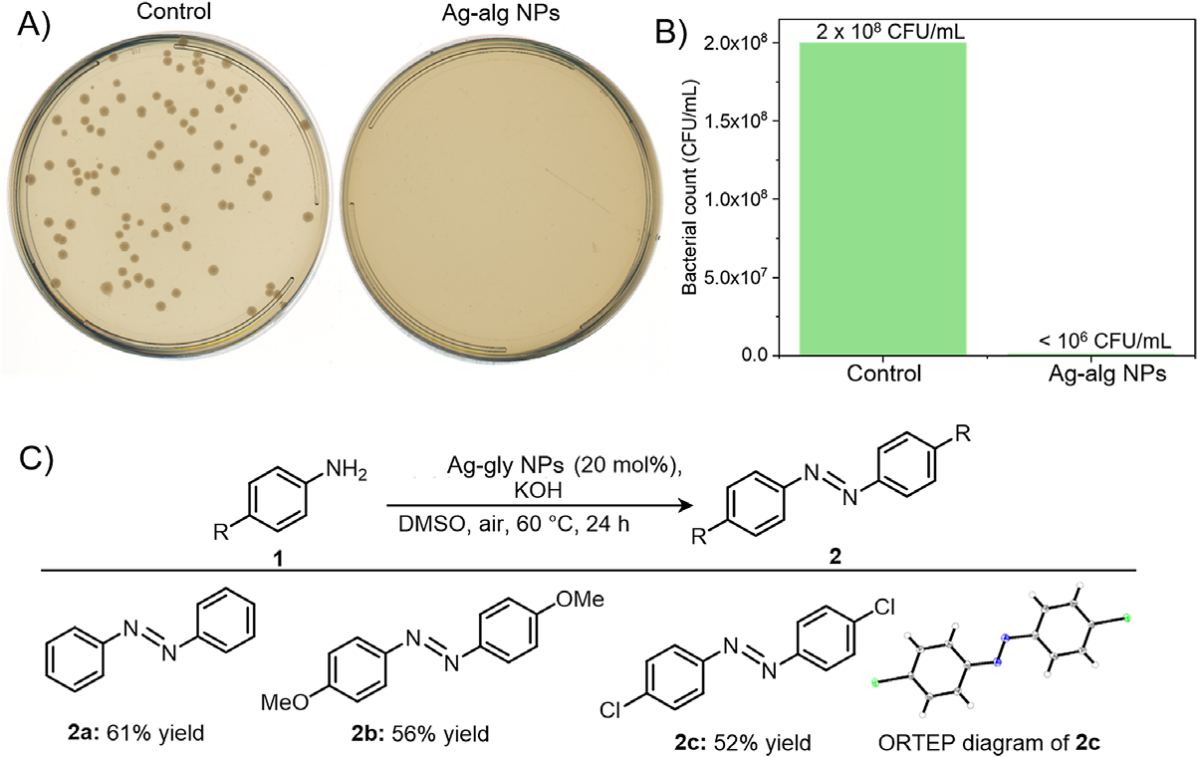
A) Photograph of the agar plates with that shows the bacterial growth in the control (untreated) and Ag-alg NPs treated sample. B) Bacterial count of control and silver nanoparticle treated samples. C) Scheme showing Ag-gly NPs catalyzed oxidative coupling of anilines. All reactions were performed on a 0.20 mmol scale for 24 h.

We conducted experiments to show the antibacterial activity of our synthesized Ag NPs. Frozen cultures of *Escherichia Coli* were refreshed on a nutrient agar medium and kept in an incubator at 37 °C for 24 h. With the help of a sterilized wire loop, a single colony from the cultures was introduced to the 20 mL nutrient medium in a 50 mL centrifuge tube and kept for 24 h in a shaker incubator at 100 rotations per minute (RPM). The broth cultures were washed with phosphate-buffered saline (PBS) and diluted to an optical density (OD)@ 600 nm = 0.1 which approximately contains 1 × 10^8^ colony forming units (CFU) per mL for further experiments. The broth cultures were further diluted to 10^6^ times for *in vitro* antibacterial testing (Ovais et al., 2022). The bacterial strain *E. Coli* was divided into two parts, one for control (no treatment) and another for the Ag-alg treatment. For *in vitro* antibacterial evaluation, the as-synthesized Ag-alg NPs were diluted 10 times and 100 μL was added to the medium containing bacteria (10 mL). As per the inductively coupled plasma mass spectrometry (ICP MS) data, 100 μL of Ag-alg NPs solution contains 13 μg of Ag. After the addition of 100 μL of Ag-alg NPs to the medium it was slightly shaken and kept for 5 min. Then, 400 μL of the treated sample and control were dispensed to the nutrient agar plates and a uniform layer of bacteria was prepared using sterilized bent glass rods. The plates were incubated at 37 °C for 24 h and finally mature colonies were counted. Figure 3A displays the growth of bacterial colonies in the control sample whereas no bacterial growth was observed in the Ag-alg NPs treated sample. This suggests that our synthesized Ag-alg NPs inhibit the growth of bacteria. When the bacteria were exposed to Ag-alg NPs, a reduction in bacterial count from 2 × 10^8^ CFU/mL to less than 10^6^ was seen Figure 3B) suggesting that the antibacterial activity is greater than 99%.

Ag NPs continuously release silver ions which may be the reason for killing bacteria. Due to strong electrostatic interaction and affinity for sulfur proteins, silver ions adhere to the cell wall and cytoplasmic membrane. The attached ions can increase the permeability of the cytoplasmic membrane and disrupt the bacterial cell. Uptake of silver ions causes deactivation of the respiratory enzymes and generates reactive oxygen species (ROS) that provoke the cell membrane disruption and deoxyribonucleic acid (DNA) modifications. As sulfur and phosphorus are important components of DNA, interaction with silver ions causes difficulties in cell production and DNA replication which results in killing the bacteria (Yin et al., 2020).

There are many other uses of silver nanoparticles in addition to their antimicrobial activity. For example, it is known that silver nanoparticles can enhance the Raman scattering cross-sections of adsorbed molecules by millions of times (Han et al., 2021).

Another exciting application is the use of silver nanoparticles to catalyze reactions, and we have utilized the *in situ*-generated silver nanoparticles for oxidative coupling reactions. In organic chemistry, N-N bond formation reactions are very important owing to the wide applications of azo compounds in biological labeling and the dye industry (Addy et al., 2017; Gürses et al., 2016). Along this line, we used our silver nanoparticles as catalysts for the oxidative coupling of anilines to form azo benzenes. With 20 mol % of silver nanoparticles as a catalyst, aniline was converted to azobenzene in 61% yield at 60 °C (Figure 3C). The N-N bond formation products were identified using NMR, IR, and Mass spectral analysis (Figure S10–S18). Furthermore, the scope of the catalyst was expanded to electron-rich 4-methoxy aniline and electron-deficient 4-chloro aniline, which afforded their corresponding oxidative coupling product in 56% and 52% yields, respectively. The structure of **2c** was further determined through X-ray crystallography (CCDC No. 2340058).

### (e) Effect of pH on the Ag NPs formation

**Figure 4.**
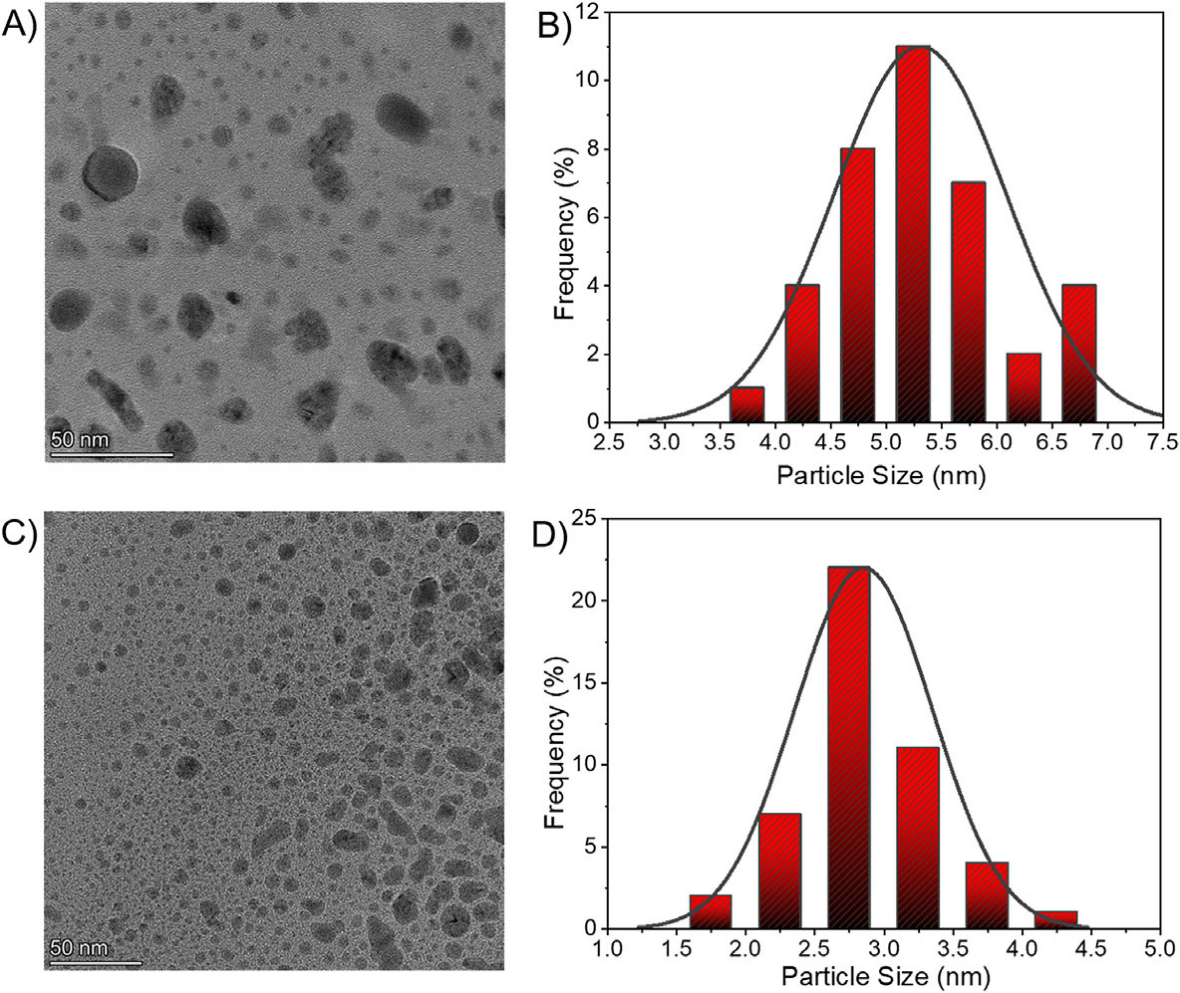
A) TEM image of the Ag NPs at pH 5. B) Size distribution of the Ag NPs at pH 5. C) TEM image of the Ag NPs at pH 10. D) Size distribution of the Ag NPs at pH 10.

To study the impact of pH on the nanoparticle formation, two different precursor solutions at pH 5 and 10 were prepared. Under acidic conditions (pH 5) the average size of the nanoparticles increases to 5.3 nm (Figure 4A and 4B) in comparison to the precursor solution prepared at neutral pH (2.9 nm shown in Figure 2B). Under basic conditions (pH 10), the average size reduces to 2.7 nm (Figure 4C and 4D). When the solution is acidic, the H^+^ ions will protonate the glycerolate oxygen atom which makes it difficult for surface functionalization of Ag NPs. As a result, the Ag NPs produced in the droplets coalesce to form larger nanoparticles. The smaller size under basic conditions may be caused by the faster deportation of glycerol protons.

### (f) Sustainability of our method

Spray using our portable nebulizing device is a simple, direct, and one-step method for producing NPs. The synthetic method is one pot and does not require extreme reaction conditions such as high temperature or pressure. The synthesis involves water as a solvent and avoids the use of toxic organic solvents. Because the method uses microdroplets for material production, the required quantity of solvent (water) is less than that of the solution phase. Besides, our method minimizes the amounts of chemical reagents required for the synthesis and does not involve the use of hazardous reducing agents such as formaldehyde, ethylene glycol, NaBH_4_, LiAlH_4_, N_2_H_4_, and so forth Single-step synthesis ensures less wastage of materials, unlike a multistep process where the processing involves the wastage of solvent and other chemicals. Direct deposition on the area of choice, fast reaction time, and ambient processing further add to the sustainability of our approach over traditional methods.

### (g) Cost analysis

**Figure 5.**
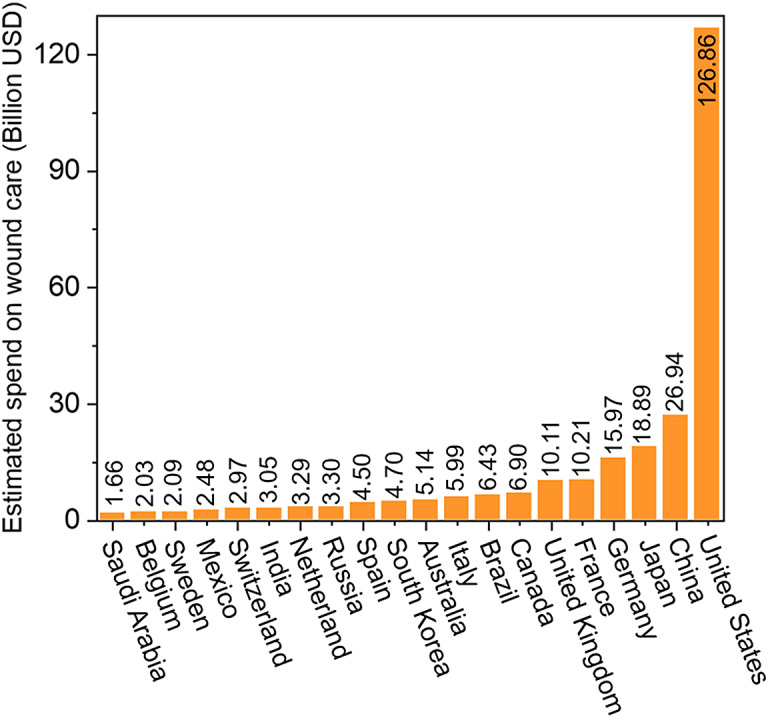
Estimated wound care cost by top 20 countries around the world in 2019 (Queen et al., 2024).

In the year 2022, nearly 10.5 million people in the United States were affected by chronic wounds (Sen, 2023). Chronic wounds are impacting the quality of life of nearly 3% of the total population of the United States. These wounds create several complications and increase the cost of health care. As per the 2019 data, the estimated U.S. expenditure for chronic wound care is 127 billion dollars per year (Queen and Harding, 2023). The second highest expenditure is 26 billion dollars by China which is 5 times less than that of the U.S. (Figure 5). As per the latest data, the U. S. expenditure on chronic wounds increased to 148 billion dollars in 2022 (Queen and Harding, 2024), a 16.5% increase in just 3 years. Affordable alternatives are needed.

We have performed a rough cost–benefit analysis to show the affordability of our method over existing ones (see supporting information). For the 10.5 million people in the US who need wound care treatment, we estimate the total cost for wound healing is approximately $30 per person which is significantly lower than the previously estimated cost of $1,085 per person.

## Conclusions

In summary, we report the ultrafast synthesis of silver nanoparticles in aqueous microdroplets at room temperature using a simple handheld mesh nebulizer sprayer. This device is easy to operate, affordable, and useful not only for the *in-situ* generation of Ag NPs but also as a portable antimicrobial sprayer, which could reduce the cost of wound dressing. The Ag NPs obtained are ∼2.9 nm in size, which shows efficacious antimicrobial activity against *E. coli* disinfecting more than 99% of bacteria. Furthermore, we also utilized Ag NPs for making azobenzenes from anilines, with up to 61% yield. Our future work will include a detailed investigation and *in vivo* analysis of animal models to assess the safety and efficacy of our spray-produced Ag NPs before clinical trials.

## Supporting information

Naveen et al. supplementary material Naveen et al. supplementary material

## Supplementary material

The supplementary material for this article can be found at http://doi.org/10.1017/qrd.2024.9.
