# Supplementary material for: Handheld portable device for delivering capped silver nanoparticles for antimicrobial applications
Source: QRB Discov. 2024 Dec 3;5:e9. doi: 10.1017/qrd.2024.9 (PMC11649374; doi:10.1017/qrd.2024.9)
Supplement: Naveen et al. supplementary material [file S2633289224000097sup001.docx]

Supporting Information for

Handheld Portable Device for Delivering Capped Silver Nanoparticles for Antimicrobial Applications

Kumar Naveen^1^, Sandeep Bose^2^, Chanbasha Basheer^3,^*, Richard N. Zare^2,^*, Elumalai Gnanamani^1,^*

^1^ Department of Chemistry, Indian Institute of Technology Roorkee, Roorkee 247667, India

^2^ Department of Chemistry, Stanford University, Stanford, CA 94305, United States

^3^ Chemistry Department, King Fahd University of Petroleum and Minerals, Dhahran 31261, Saudi Arabia

* Corresponding authors. Email: cbasheer@kfupm.edu.sa; zare@stanford.edu; gnanam@cy.iitr.ac.in

Summary: 22 pages, 19 figures, 11 tables .

**Synthesis of Nickel nanoparticles:**

10 mM of nickel acetate [Ni(CH_3_COO)_2_.4H_2_O] was prepared in milli-q water. The aqueous solution of Ni(CH_3_COO)_2_.4H_2_O was directly sprayed on top of a TEM grid for 30 s to check the nanoparticle formation.

**Figure S1.** Photographic image of the mess nebulizer sprayer producing microdroplets.

**Figure S2.** Photographic image of the reactant mixture before and after spray. The brown color after spray indicates the formation of Ag NPs.

**Figure S3.** Energy dispersive X-ray (EDS) measurement showing the presence of Ag in the droplet-synthesized nanoparticles.

**Figure S4.** TEM image of the silver nanoparticles obtained without using glycerol and sodium alginate.

**Figure S5**. C 1s peak of A) Ag-Alg NPs and B) sodium alginate.

**Figure S6**. A) TEM image of the Ag-Alg NPs. B) EDS spectrum of the Ag-Alg NPs. C) XPS feature showing Ag 3d peaks. D) XRD of Ag-Alg NPs.

**Figure S7.** A) TEM image of the nickel nanoparticles. B) EDS spectrum of nickel nanoparticles.

**Figure S8**. TEM (A-F) and SEM (G&H) images showing nanoparticles and their respective size distribution plot obtained by using different spray technique.

**Figure S9.** A comparison of average droplet size generated by different spray technique.

**General procedure 1: Ag-glycerate nanoparticles catalyzed azo-coupling of aniline.**

The synthesis of azobenzenes were carried out according to a previous literature report.^4^ An oven-dried 10 ml round bottom flask was charged with silver-glycerate nanoparticles (0.2 equiv., 0.04 mmol), followed by the addition of aniline (1.0 equiv., 0.2 mmol) and potassium hydroxide (KOH) (1.0 equiv., 0.2 mmol). The resulting mixture was dissolved in dimethyl sulfoxide (DMSO) (0.4 ml) and stirred at 60 °C for 24 h under air (1 atm). Reaction progress was monitored by TLC. After cooling down to room temperature, the mixture was purified by flash column chromatography on a short silica gel (eluent: 100% hexane → 5:95 hexane/EtOAc) to afford the desired compound.

**Table S1.** Optimization of reaction conditions.*^a^*

| **S.No.** | **Ag-gly nanoparticles (mol%)** | **Time (h)** | **Yield (%)*^b^*** |
| --- | --- | --- | --- |
| 1 | 10 | 40 | 50 |
| 2 | 20 | 24 | 61 |
| 3 | 10 | 24 | 46 |

*^a^*Reactions were carried out by following the general procedure. *^b^*Isolated yields.

**(*E*)-1,2-diphenyldiazene (2a)**

The title compound was prepared according to the general procedure-3 using aniline (18.6 mg, 1.0 equiv., 0.2 mmol), silver- glycerate nanoparticles (7.9 mg, 0.2 equiv., 0.04 mmol), and potassium hydroxide (11.3 mg, 1.0 equiv., 0.2 mmol) in DMSO (0.4 mL). Purification by column chromatography (100:0 → 95:5 hexane/EtOAc) afforded the title compound as an orange solid. (11.2 mg, 61% yield) The ^1^H, ^13^C NMR and FT-IR data match the literature.^4^

**Physical appearance:** Orange Solid

**Melting Point:** 67-68 °C (lit. 66-68 °C)^5^

**^1^H NMR (CDCl_3_, 500 MHz), ppm:** δ 7.96 – 7.91 (m, 4H), 7.56 – 7.47 (m, 6H).

**^13^C NMR (CDCl_3_, 126 MHz), ppm:** δ 152.8, 131.1, 129.2, 122.9.

**FT-IR (neat, cm^-1^):** 3063, 2346, 1956, 1482, 1453 (N=N), 1299, 12210, 1151, 1071, 776, 690.

**HRMS-ESI (*m/z*):** Calculated for C_12_H_11_N_2_^+^ [M+H]: 183.0917, Found [M+H]: 183.0916.

**(*E*)-1,2-****bis(4-methoxyphenyl)diazene (2b)**

The title compound was prepared according to the general procedure-3 using 4-methoxy aniline (24.6 mg, 1.0 equiv., 0.2 mmol), silver-glycerate nanoparticles (7.9 mg, 0.2 equiv., 0.04 mmol), and potassium hydroxide (11.3 mg, 1.0 equiv., 0.2 mmol) in DMSO (0.4 mL). Purification by column chromatography (97:3 → 92:8 hexane/EtOAc) afforded the title compound as yellow solid. (13.5 mg, 56% yield) The ^1^H, ^13^C NMR and FT-IR data match the literature.^6^

**Physical appearance:** Yellow Solid

**Melting Point:** 155-156 °C (lit. 154.6-156.3 °C)^5^

**^1^H NMR (CDCl_3_, 500 MHz), ppm:** δ 7.88 (d, *J* = 9.0 Hz, 4H), 7.00 (d, *J* = 9.0 Hz, 4H), 3.89 (s, 6H).

**^13^C NMR (CDCl_3_, 126 MHz), ppm:** δ 161.7, 147.2, 124.5, 114.3, 55.7.

**FT-IR (neat, cm^-1^):** 2930, 2858, 1601, 1576, 1495 (N=N), 1243 (C-O), 1022 (C-O), 840, 540.

**HRMS-ESI (*m/z*):** Calculated for C_14_H_15_N_2_O_2_^+^ [M+H]: 243.1128, Found [M+H]: 243.1118.

**(*E*)-1,2-bis(4-chlorophenyl)diazene (2c)**

The title compound was prepared according to the general procedure-3 using 4-chloro aniline (25.5 mg, 1.0 equiv., 0.2 mmol), silver-glycerate nanoparticles (7.9 mg, 0.2 equiv., 0.04 mmol), and potassium hydroxide (11.3 mg, 1.0 equiv., 0.2 mmol) in DMSO (0.4 mL). Stir the reaction mixture for 36 h. Purification by column chromatography (97:3 → 92:8 hexane/EtOAc) afforded the title compound as an orange solid. (13 mg, 52% yield) The ^1^H, ^13^C NMR and FT-IR data match the literature.^4,7^

**Physical appearance:** Orange Solid

**Melting Point:** 178-179 °C (lit. 178.7-181.6 °C)^8^

**^1^H NMR (CDCl_3_, 500 MHz), ppm:** δ 7.87 (d, *J* = 8.7 Hz, 4H), 7.49 (d, *J* = 8.7 Hz, 4H).

**^13^C NMR (CDCl_3_, 126 MHz), ppm:** δ 150.9, 137.4, 129.6, 124.3.

**FT-IR (neat, cm^-1^):** 2930, 2860, 1571, 1477 (N=N), 1404, 1081, 1001, 842 (C-Cl), 648.

**HRMS-ESI (*m/z*):** Calculated for C_12_H_9_Cl_2_N_2_^+^ [M+H]: 251.0137, Found [M+H]: 251.0127.

**
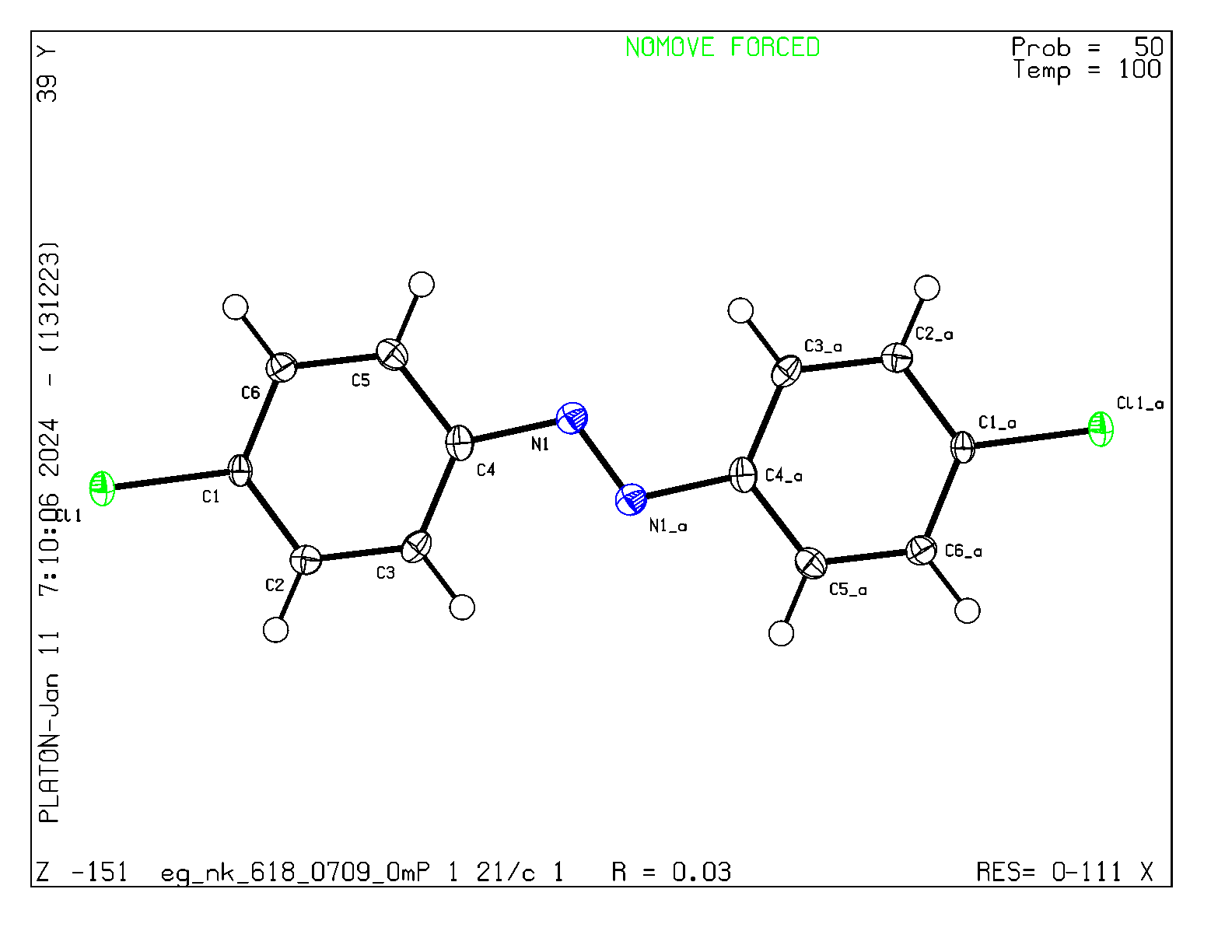
Crystal Structure Information – EG_NK_618_0709_0m_a**

| **Table S2 Crystal data and structure refinement for EG_NK_618_0709_0m_a.** | |
| --- | --- |
| Identification code | EG_NK_618_0709_0m_a |
| Empirical formula | C_12_Cl_2_N_2_H_8_ |
| Formula weight | 251.11 |
| Temperature/K | 100.00 |
| Crystal system | monoclinic |
| Space group | P2_1_/c |
| a/Å | 9.7698(6) |
| b/Å | 4.6678(3) |
| c/Å | 11.4529(7) |
| α/° | 90 |
| β/° | 91.067(2) |
| γ/° | 90 |
| Volume/Å^3^ | 522.20(6) |
| Z | 4 |
| ρ_calc_g/cm^3^ | 1.597 |
| μ/mm^‑1^ | 0.589 |
| F(000) | 256.0 |
| Crystal size/mm^3^ | 0.685 × 0.093 × 0.015 |
| Radiation | MoKα (λ = 0.71073) |
| 2Θ range for data collection/° | 4.17 to 56.606 |
| Index ranges | -13 ≤ h ≤ 13, -6 ≤ k ≤ 6, -15 ≤ l ≤ 15 |
| Reflections collected | 13473 |
| Independent reflections | 1298 [R_int_ = 0.0510, R_sigma_ = 0.0281] |
| Data/restraints/parameters | 1298/0/73 |
| Goodness-of-fit on F^2^ | 1.052 |
| Final R indexes [I>=2σ (I)] | R_1_ = 0.0302, wR_2_ = 0.0773 |
| Final R indexes [all data] | R_1_ = 0.0314, wR_2_ = 0.0784 |
| Largest diff. peak/hole / e Å^-3^ | 0.50/-0.23 |

| **Table S3 Fractional Atomic Coordinates (×10^4^) and Equivalent Isotropic Displacement Parameters (Å^2^×10^3^) for EG_NK_618_0709_0m_a. U_eq_ is defined as 1/3 of the trace of the orthogonalised U_IJ_ tensor.** | | | | |
| --- | --- | --- | --- | --- |
| **Atom** | ***x*** | ***y*** | ***z*** | **U(eq)** |
| Cl1 | 9178.3(3) | 827.0(7) | 3321.4(3) | 14.88(13) |
| N1 | 5451.3(12) | 9469(2) | 5333.6(10) | 15.0(2) |
| C1 | 8055.5(12) | 3322(3) | 3893.1(11) | 11.8(3) |
| C2 | 7025.1(14) | 4430(3) | 3174.4(11) | 13.9(3) |
| C3 | 6139.8(13) | 6473(3) | 3613.1(12) | 14.9(3) |
| C4 | 6289.0(13) | 7371(3) | 4777.3(11) | 14.3(3) |
| C5 | 7313.7(14) | 6189(3) | 5485.6(12) | 15.5(3) |
| C6 | 8206.4(14) | 4161(3) | 5049.0(12) | 14.9(3) |

| **Table S4 Anisotropic Displacement Parameters (Å^2^×10^3^) for EG_NK_618_0709_0m_a. The Anisotropic displacement factor exponent takes the form: -2π^2^[h^2^a*^2^U_11_+2hka*b*U_12_+…].** | | | | | | |
| --- | --- | --- | --- | --- | --- | --- |
| **Atom** | **U_11_** | **U_22_** | **U_33_** | **U_23_** | **U_13_** | **U_12_** |
| Cl1 | 12.82(18) | 14.68(18) | 17.29(19) | -0.63(11) | 3.78(12) | 2.8(1) |
| N1 | 12.9(5) | 16.2(5) | 16.0(5) | 0.7(4) | 0.7(4) | -0.9(4) |
| C1 | 9.5(5) | 11.9(6) | 14.2(6) | 0.4(5) | 3.9(4) | 0.4(5) |
| C2 | 12.5(6) | 15.6(6) | 13.7(6) | 0.3(5) | 0.4(5) | -2.0(5) |
| C3 | 10.7(6) | 16.2(6) | 17.7(6) | 3.3(5) | -0.2(5) | 0.7(5) |
| C4 | 11.9(6) | 13.4(6) | 17.9(6) | 0.6(5) | 4.9(5) | -0.9(5) |
| C5 | 16.1(6) | 16.6(6) | 14.0(6) | -1.1(5) | 2.5(5) | -1.2(5) |
| C6 | 13.6(6) | 17.0(6) | 14.0(6) | 1.1(5) | 0.2(5) | 0.2(5) |

| **Table S5 Bond Lengths for EG_NK_618_0709_0m_a.** | | | | | | |
| --- | --- | --- | --- | --- | --- | --- |
| **Atom** | **Atom** | **Length/Å** |  | **Atom** | **Atom** | **Length/Å** |
| Cl1 | C1 | 1.7366(13) |  | C2 | C3 | 1.3879(19) |
| N1 | N1^1^ | 1.258(2) |  | C3 | C4 | 1.4027(19) |
| N1 | C4 | 1.4331(17) |  | C4 | C5 | 1.3908(19) |
| C1 | C2 | 1.3883(18) |  | C5 | C6 | 1.3869(19) |
| C1 | C6 | 1.3857(18) |  |  |  |  |

^1^1-X,2-Y,1-Z

| **Table S6 Bond Angles for EG_NK_618_0709_0m_a.** | | | | | | | | |
| --- | --- | --- | --- | --- | --- | --- | --- | --- |
| **Atom** | **Atom** | **Atom** | **Angle/˚** |  | **Atom** | **Atom** | **Atom** | **Angle/˚** |
| N1^1^ | N1 | C4 | 113.52(14) |  | C3 | C4 | N1 | 125.22(12) |
| C2 | C1 | Cl1 | 118.84(10) |  | C5 | C4 | N1 | 114.99(11) |
| C6 | C1 | Cl1 | 119.54(10) |  | C5 | C4 | C3 | 119.79(12) |
| C6 | C1 | C2 | 121.63(12) |  | C6 | C5 | C4 | 120.74(12) |
| C3 | C2 | C1 | 119.41(12) |  | C1 | C6 | C5 | 118.74(12) |
| C2 | C3 | C4 | 119.68(12) |  |  |  |  |  |

^1^1-X,2-Y,1-Z

| **Table S7 Torsion Angles for EG_NK_618_0709_0m_a.** | | | | | | | | | | |
| --- | --- | --- | --- | --- | --- | --- | --- | --- | --- | --- |
| **A** | **B** | **C** | **D** | **Angle/˚** |  | **A** | **B** | **C** | **D** | **Angle/˚** |
| Cl1 | C1 | C2 | C3 | -178.58(10) |  | C2 | C1 | C6 | C5 | -1.0(2) |
| Cl1 | C1 | C6 | C5 | 179.00(10) |  | C2 | C3 | C4 | N1 | 179.63(12) |
| N1^1^ | N1 | C4 | C3 | -1.3(2) |  | C2 | C3 | C4 | C5 | -0.6(2) |
| N1^1^ | N1 | C4 | C5 | 178.92(14) |  | C3 | C4 | C5 | C6 | 1.0(2) |
| N1 | C4 | C5 | C6 | -179.18(12) |  | C4 | C5 | C6 | C1 | -0.2(2) |
| C1 | C2 | C3 | C4 | -0.6(2) |  | C6 | C1 | C2 | C3 | 1.4(2) |

^1^1-X,2-Y,1-Z

| **Table S8 Hydrogen Atom Coordinates (Å×10^4^) and Isotropic Displacement Parameters (Å^2^×10^3^) for EG_NK_618_0709_0m_a.** | | | | |
| --- | --- | --- | --- | --- |
| **Atom** | ***x*** | ***y*** | ***z*** | **U(eq)** |
| H2 | 6926.81 | 3794.58 | 2389.23 | 17 |
| H3 | 5436.22 | 7259.04 | 3127.35 | 18 |
| H5 | 7403.21 | 6778.24 | 6277.39 | 19 |
| H6 | 8907.94 | 3361.07 | 5533.22 | 18 |

**Experimental**

Single crystals of C_6_ClNH_4_ **[EG_NK_618_0709_0m_a]** were **[]**. A suitable crystal was selected and **[]** on a **Bruker APEX-II CCD** diffractometer. The crystal was kept at 100.00 K during data collection. Using Olex2^9^, the structure was solved with the XT^10^ structure solution program using Intrinsic Phasing and refined with the XL^11^ refinement package using Least Squares minimization.

**Crystal structure determination of [EG_NK_618_0709_0m_a]**

**Crystal Data** for C_6_ClNH_4_ (*M*=125.55 g/mol): monoclinic, space group P2_1_/c (no. 14), *a* = 9.7698(6) Å, *b* = 4.6678(3) Å, *c* = 11.4529(7) Å, *β* = 91.067(2)°, *V*= 522.20(6) Å^3^, *Z* = 4, *T* = 100.00 K, μ(MoKα) = 0.589 mm^-1^, *Dcalc* = 1.597 g/cm^3^, 13473 reflections measured (4.17° ≤ 2Θ ≤ 56.606°), 1298 unique (*R*_int_ = 0.0510, R_sigma_ = 0.0281) which were used in all calculations. The final *R*_1_ was 0.0302 (I > 2σ(I)) and *wR*_2_ was 0.0784 (all data).

**Refinement model description**

Number of restraints - 0, number of constraints - unknown.

Details:

1. Fixed Uiso
 At 1.2 times of:
 All C(H) groups
2.a Aromatic/amide H refined with riding coordinates:
 C2(H2), C3(H3), C5(H5), C6(H6)

**Spectral data of coupling products:**

|   **Figure S10: ^1^H NMR spectrum of (*E*)-1,2-diphenyldiazene (2a)** |
| --- |
|   **Figure S11: ^13^C NMR spectrum of (*E*)-1,2-diphenyldiazene (2a)** |

| 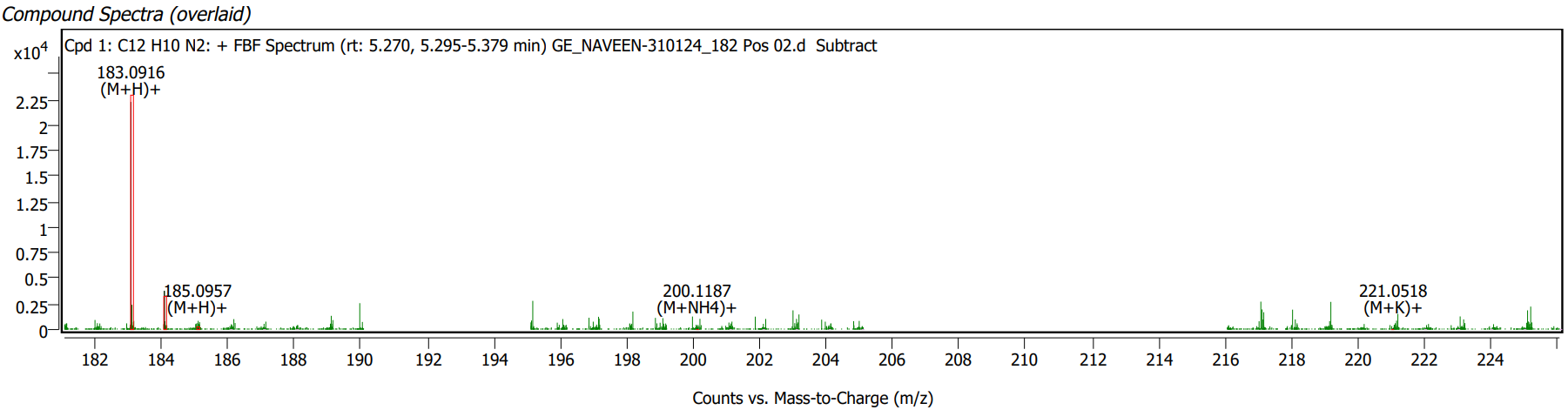  **Figure S12: HRMS spectrum of (*E*)-1,2-diphenyldiazene (2a)** |
| --- |

|   **Figure S13:^1^H NMR spectrum of (*E*)-1,2-bis(4-methoxyphenyl)diazene (2b** |
| --- |
|   **Figure S14:^13^C NMR spectrum of (*E*)-1,2-bis(4-methoxyphenyl)diazene (2b)** |
| 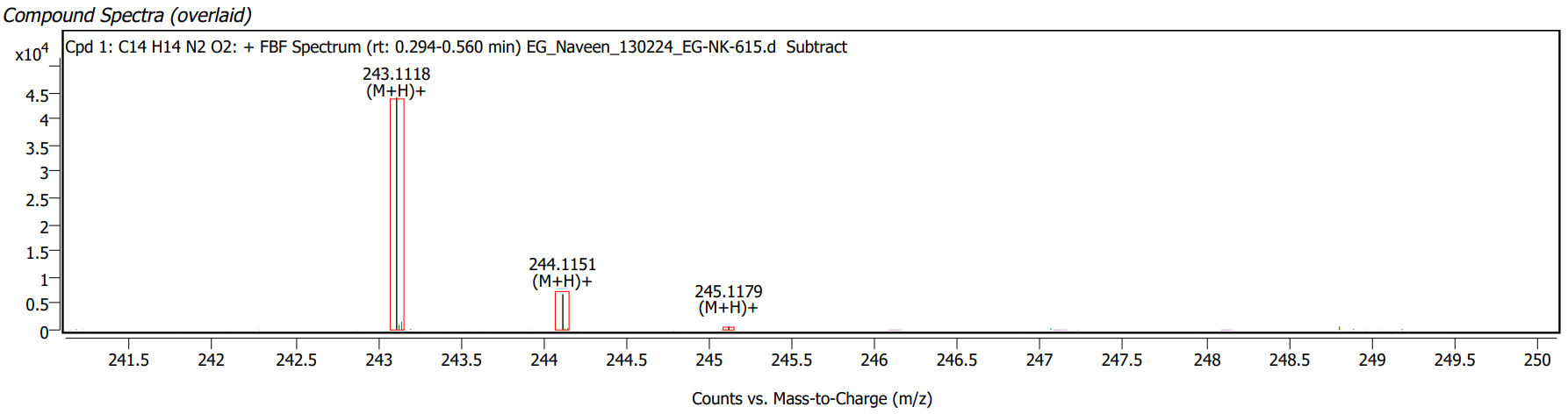  **FigureS15: HRMS spectrum of (*E*)-1,2-bis(4-methoxyphenyl)diazene (2b)** |

|   **Figure S16: ^1^H NMR spectrum of (*E*)-1,2-bis(4-chlorophenyl)diazene (2c)** |
| --- |
|   **Figure S17: ^13^C NMR spectrum of ((*E*)-1,2-bis(4-chlorophenyl)diazene (2c)** |

| 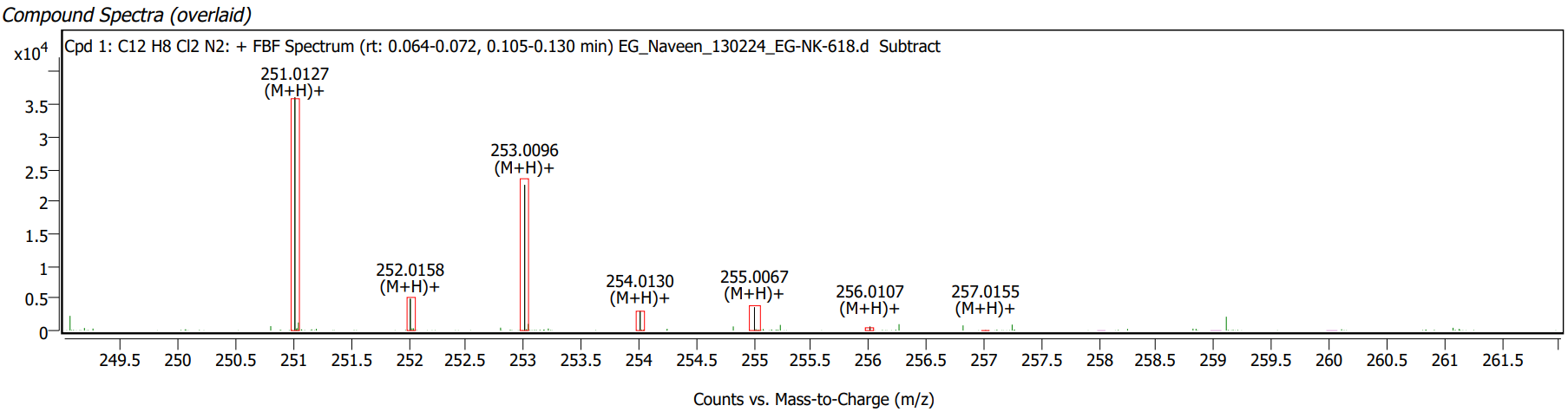  **Figure S18: HRMS spectrum of (*E*)-1,2-bis(4-chlorophenyl)diazene (2c)** |
| --- |

**Detailed Cost calculation**:

As per our calculation, the average money spent for chronic wounds treatment is U.S. is $ 14,095 per person (Table S9).^1,2^ We know that the cost includes the facility, physicians, home nursing, dressing, HBOT (hyperbaric oxygen therapy), NPWT (negative pressure wound therapy), and bio-skin.^3^ We are here more focused on the wound dressing aspect as our synthesized Ag NPs will be useful for wound dressing. Out of all the major cost drivers, dressing contributes 7.7 % of the total cost required for wound healing (Figure S18). As per calculation, the cost of wound dressing per person is $ 1,085 (7.7 % of $ 14,095) (Table S10). Our prototype produces microdroplets at a flow rate of 600 µL/min. Assuming each time the treatment requires 30 s spray on the wound for healing and complete healing a minimum of 10 sprays (1 spray each for 10 days) is required. The total time required to spray for complete healing is (10 x 30 s) 300 s for which we need 3 mL of the precursor solution for spray. For our Ag NPs synthesis, 0.025 mmol of AgNO_3_ (4.95 mg) and 0.025 mmol (4.25 mg) of sodium alginate were used to prepare a 3 mL mixture solution. As we discussed earlier, a total of 3 mL of precursor solution is required for complete healing, and the total cost of material (4.95 mg of AgNO_3_ + 4.25 mg of sodium alginate + water) to prepare 3 mL of solution is estimated to be $ 1.015 (Table S11). Additional cost includes device charges ($ 15), labor charges ($ 8), power consumption ($ 4), and adhesive bandage ($ 2) to prevent contamination. Thus, the total cost for wound healing is approximately $ 30 per person which is significantly lower than the previously estimated cost of $1,085 per person. The amount saved per person is $1055. For 10.5 million people we could save approximately a whopping $ 11.07 billion and provide affordable health care for everyone. The estimated cost may slightly vary depending on the type of wound and the time it takes to heal the wound.

| No. of people affected by chronic wound in U.S. in 2022 = 10.5 million^1^ |
| --- |
| Estimated spend on wound care in 2022 = $ 148 billion^2^ |
| The average money spend on wound care per person = $148 billion/10.5 million = $14,095 |

**Table S9.** The cost of wound care per person in U.S.^1,2^

**Figure S19.** Breakdown of cost to heal wounds by various factors.^3^

**Table S10.** Breakdown cost for wound healing per person by various categories.^3^

| **Categories** | **% contribution in wound healing** | **Contribution** **in $ (per person)** |
| --- | --- | --- |
| Facility | 30 | 4228 |
| Physician | 12.5 | 1762 |
| Dressing | 7.7 | 1085 |
| Home health | 18.4 | 2593 |
| HBOT | 17.9 | 2523 |
| NPWT | 13.1 | 1846 |
| Bioskin | 0.4 | 56 |

**Table S11.** Cost-benefit analysis of using our method for antimicrobial wound healing.

| **Categories** | **Cost in $ (per person)** |
| --- | --- |
| Materials  (4.95 mg of AgNO_3_ + 4.25 mg of sodium alginate + water)  Cost of AgNO_3_ - $1520/500g, cost of sodium alginate - $150/Kg | 1.015 |
| Ultrasonic device | 15 |
| Power consumption | 4 |
| Labor | 8 |
| Adhesive bandage | 2 |
| **Total cost for wound healing** | **~ 30** |
| Previously literature based estimated cost of wound healing | 1085 |
| Cost benefit = Previous estimated cost - Our estimated cost | 1055 |
| **Total saving for 10.5 million people = 10.5 million x 1055** | **~ 11 billion** |

**References**

(1) Sen, C. K. Human Wound and Its Burden: Updated 2022 Compendium of Estimates. *Adv. wound care* **2023**, *12* (12), 657–670. https://doi.org/10.1089/wound.2023.0150.

(2) Queen, D.; Harding, K. Estimating the Cost of Wounds Both Nationally and Regionally within the Top 10 Highest Spenders. *Int. Wound J.* **2024**, *21* (2), e14709. https://doi.org/10.1111/iwj.14709.

(3) Fife, C. E.; Carter, M. J. Wound Care Outcomes and Associated Cost Among Patients Treated in US Outpatient Wound Centers: Data From the US Wound Registry. *Wounds a Compend. Clin. Res. Pract.* **2012**, *24* (1), 10–17.

(4) Cai, S.; Rong, H.; Yu, X.; Liu, X.; Wang, D.; He, W.; Li, Y. Room Temperature Activation of Oxygen by Monodispersed Metal Nanoparticles: Oxidative Dehydrogenative Coupling of Anilines for Azobenzene Syntheses. *ACS Catal.* **2013,** *3* (4), 478–486.

(5) Kaur, L.; Kumar, M.; Bhalla, V. Type I Strong Acceptor–Weak Acceptor Photosensitizing Assemblies for the Regulated Aerobic Oxidative Coupling of Anilines. *Green Chem.* **2023,** *25* (13), 5240–5246.

(6) Zhang, C.; Jiao, N. Copper‐catalyzed Aerobic Oxidative Dehydrogenative Coupling of Anilines Leading to Aromatic Azo Compounds Using Dioxygen as an Oxidant. *Angew. Chem. Int. Ed Engl.* **2010,** *49* (35), 6174–6177.

(7) Dey, S.; Panja, D.; Sau, A.; Thakur, S. D.; Kundu, S. Reusable Cobalt-Catalyzed Selective Transfer Hydrogenation of Azoarenes and Nitroarenes. *J. Org. Chem.* **2023,** *88* (14), 10048–10057.

(8) Cepanec, I.; Litvić, M.; Udiković, J.; Pogorelić, I.; Lovrić, M. Copper(I)-Catalysed Homo-Coupling of Aryldiazonium Salts: Synthesis of Symmetrical Biaryls. *Tetrahedron* **2007,** *63* (25), 5614–5621.

(9) Dolomanov, O. V.; Bourhis, L. J.; Gildea, R. J.; Howard, J. A. K.; Puschmann, H. OLEX2: A Complete Structure Solution, Refinement, and Analysis Program. *J. Appl. Cryst.* **2009,** *42,* 339-341.

(10) Sheldrick, G. M. SHELXT - Integrated Space-Group and Crystal-Structure Determination. *Acta Cryst.* **2015,** *A71*, 3-8.

(11) Sheldrick, G. M. A Short History of SHELX. *Acta Cryst.* **2008,** *A64*, 112.
