# Supplementary material for: Handheld portable device for delivering capped silver nanoparticles for antimicrobial applications
Source: QRB Discov. 2024 Dec 3;5:e9. doi: 10.1017/qrd.2024.9 (PMC11649374; doi:10.1017/qrd.2024.9)
Supplement: Peer review history

# Editor report: Decision: Handheld portable device for delivering capped silver nanoparticles for antimicrobial applications — R0/PR2

**DOI:** 10.1017/qrd.2024.9.pr2

**Affiliations:** Chemistry, Chalmers University of Technology, Sweden

No accompanying comment.

# Editor report: Decision: Handheld portable device for delivering capped silver nanoparticles for antimicrobial applications — R1/PR4

**DOI:** 10.1017/qrd.2024.9.pr4

**Affiliations:** Chemistry, Chalmers University of Technology, Sweden

No accompanying comment.
